# A neuronal wiring platform through microridges for rationally engineered neural circuits

**DOI:** 10.1063/5.0025921

**Published:** 2020-12-08

**Authors:** Yu Wu, Meijian Wang, Yong Wang, Huiran Yang, Hao Qi, Benjamin J. Seicol, Ruili Xie, Liang Guo

**Affiliations:** 1 Department of Electrical and Computer Engineering, The Ohio State University, Columbus, Ohio 43210, USA; 2 Department of Otolaryngology, The Ohio State University, Columbus, Ohio 43210, USA; 3 Department of Neuroscience, The Ohio State University, Columbus, Ohio 43210, USA

## Abstract

Precisely engineered neuronal circuits are promising for both fundamental research and clinical applications. However, randomly plating thousands of cells during neural network fabrication remains a major technical obstacle, which often results in a loss of tracking in neurons' identities. In this work, we demonstrated an accurate and unique neural wiring technique, mimicking neurons' natural affinity to microfibers. SU-8 microridges, imitating lie-down microfibers, were photolithographically patterned and then selectively coated with poly-l-lysine. We accurately plated *Aplysia californica* neurons onto designated locations. Plated neurons were immobilized by circular microfences. Furthermore, neurites regrew effectively along the microridges *in vitro* and reached adjacent neurons without undesirable crosstalks. Functional chemical synapses also formed between accurately wired neurons, enabling two-way transmission of electrical signals. Finally, we fabricated microridges on a microelectrode array. Neuronal spikes, stimulation-evoked synaptic activity, and putative synaptic adaption between connected neurons were observed. This biomimetic platform is simple to fabricate and effective with neurite pathfinding. Therefore, it can serve as a powerful tool for fabricating neuronal circuits with rational design, organized cellular communications, and fast prototyping.

## INTRODUCTION

Composed of hundreds^1^ to billions^2^ of wired neurons, the nervous system controls the most sophisticated functions in animals, such as movement, memory, and cognition. Although similar to electronic circuits in many ways, functional circuits composed of living neurons are still in their early exploration.^3,4^ Fabrication of such neurobiological circuits requires both micropatterning of individual neurons and accurate guidance of their neurites. Chemical patterning of biomolecules, such as microcontact printing,^5–10^ microfluidic patterning,^11–13^ laser ablation,^14–16^ and liftoff patterning,^17–22^ is an effective strategy for guiding neurites toward chemically defined directions. However, without physical constraints on cell migration, it is still challenging to immobilize a particular neuron at a specific location during the process of cell seeding. Furthermore, although single-cell resolution could be achieved with extracellular matrix (ECM) protein patterning^23^ and poly-l-lysine (PLL) surface patterning,^24^ plating thousands of neurons all together and then letting cells randomly attach to predefined adhesion spots (the rest of unattached cells are washed away) often result in a neuronal network with unknown identities of each cell, thus failing to produce a rationally and accurately defined neuronal circuit. Group cell patterning with acoustic waves^25^ or magnetic force^26^ also suffers from this issue of identity loss.

On the other hand, micromachining enables the fabrication of topographically defined culture environments in order to physically restrict and trap neurons or guide neurite regrowth. Microchannels,^27–30^ microgrooves,^31–34^ and micropillars^35,36^ are representative technologies. However, trapping cells in microwells^37^ and guiding their axons through microchannels are also problematic. It often ends up with either a low degree of neurite polarity due to large channel dimensions or uncontrolled growth cones that migrate out of the channels. Neurite guidance was also realized by the promising 3D microscaffolds.^38^ However, it is difficult to integrate 3D constructs with electrophysiology tools, such as microelectrode arrays (MEAs), to monitor and intervene neural activity. Approaches of periodic microgrooves and micropillars, utilizing local adhesion forces as guidance cues, have not been tailored for fabricating accurately wired neuronal circuits.

Rather than imposing intensive trapping forces to neuronal projections, the pathfinding and migration of *in vivo* neurons are effectively guided by highly organized fibrous structures, such as radial glia processes in the brain and the fascicular structure in peripheral nerves.^39,40^ Inspired by this fiber-assisted guidance mechanism, here we demonstrated an accurate neural wiring technique based on the finding that rat dorsal root ganglion neurons regenerate neurites along microfibers *in vitro.*^41–43^ Specifically, microfiber-like ridges (20-*μ*m wide and 25-*μ*m high) were patterned and cured on silicon nitride substrates through one-layer photolithography. We, then, selectively deposited PLL on the sidewalls of microridges using surface tension. In order to avoid cell displacements from plating, vibration, and migrations, circular SU-8 microfences with outlets were used to immobilize *Aplysia californica* neuronal somata to designated locations. Additionally, once neurons adhered to the substrate after a 24 h culture, the microfences also served as topographical cues to guide the orientation of polarized neurites. With the integrative guiding cues of the microridges and PLL, as well as the soma-anchoring microfences, we achieved both precise cell placements and accurate neurite guidance of *Aplysia californica* neurons, with minimal neurite branching. Furthermore, with simultaneous intracellular recording and extracellular stimulation, we confirmed that accurately wired neurons could fire action potentials and transmit excitatory postsynaptic potentials (EPSPs) through chemical synapses. Finally, we demonstrated with single neuron recording that stimulation-evoked activities were observed on a microelectrode array (MEA) integrated with the microridge scaffold.

## RESULTS

### Microridge fabrication

Although the guiding effect of a microfiber had been previously reported,^41^ and our proof-of-concept tests confirmed that *Aplysia californica* neurons could be guided by microfibers (Fig. S1), a great deal of engineering efforts is still required to apply this principle to fabricate a rationally designed neuronal circuit. For example, each individual fiber needs to be precisely positioned and immobilized on a substrate at microscale, which is hard to achieve with conventional electrospun microfibers. Additionally, it is also imperative to immobilize neuronal somata and minimize neurite outgrowth deviating away from the intended paths.

**FIG. 1.**
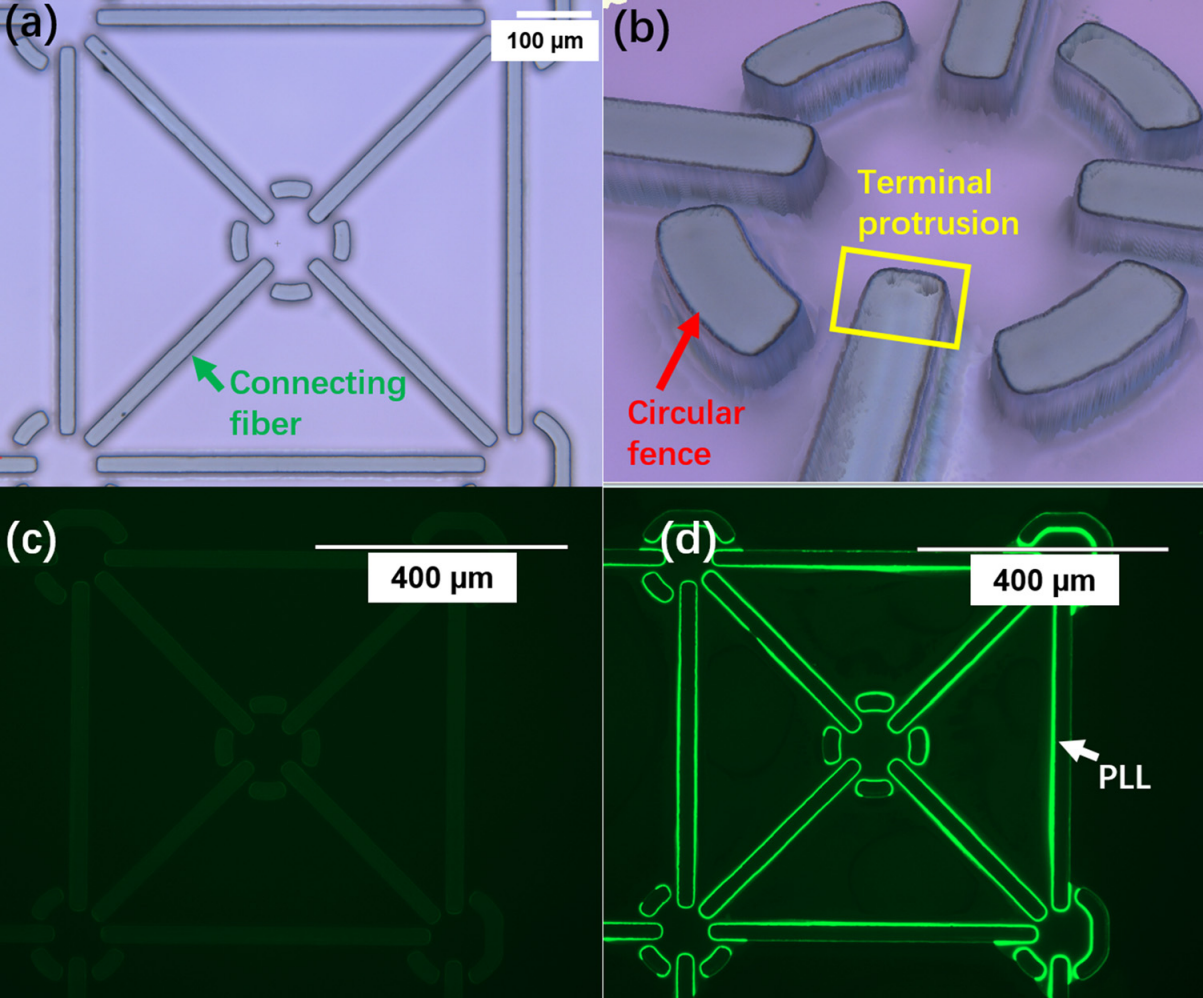
Fabricated microridge scaffold with selective PLL coating. (a) Optical microscopic image of a scaffold unit consisting of five microfences with mutual connecting lines. The scale bar is 100 *μ*m. (b) 3D reconstructed image of a microfence by a ZETA profiler, where the arcs helped to restrain the soma and provide topographical cues and the protrusions of microridge terminals helped with early stage neurite orientations. (c) Green fluorescent image of an uncoated scaffold unit as fabricated. The microridges exhibited weak and uniform autofluorescence. (d) Green fluorescent image of the scaffold unit after selective coating of FITC labeled PLL. Under the same microscopy settings, edges of the microridges exhibited strong fluorescence, indicating effective selective PLL coating. The scale bar in (c) and (d) is 400 *μ*m.

On a planar substrate, microscale ridges would be a good imitation of lie-down microfibers topographically. Here, SU-8 photoresist was chosen as our scaffold material due to its good processability, biocompatibility, and robustness in the cell culture environment. Fabrication of SU-8 microstructures had been well developed, and thus, it could be integrated with many microelectronic devices, such as MEAs for neural recording and stimulation. As shown in Fig. 1(a), 20-*μ*m microridges with a high aspect ratio could be rapidly fabricated by a single-layer photolithography process. The microfences of 25-*μ*m height, though lower than *Aplysia californica* neurons (50 *μ*m–100 *μ*m in diameter), constituted physical barriers that are sufficient to keep the somata close to the entry terminals of connecting lines. This allows the topographical cues of the microridges to be sensed by the neurons. Meanwhile, these barriers, with the same dimensions as those of the connecting line terminals, could provide guiding cues to orient the growth cones toward the microridge entry terminals at the initial stage [Fig. 1(b)].

In contrast, we observed that the directions of neurite outgrowth deviated from a connecting line when the entry terminal was at the perimeter of a circular microfence (Fig. S2), possibly due to a failure of sensing the topographical cue. Therefore, entry terminals were placed inside the perimeter to effectively guide neurite orientations at the early stages of pathfinding [Fig. 1(b)].

### Neurite guidance by the selectively coated scaffold

Due to the hydrophobicity of silicon nitride and SU-8, uncoated devices provided poor adhesion for cell attachment (leading to apoptosis) and nonselective growth cone trajectories [Fig. S3(a)]. On the other hand, uniform coating of the adhesive molecule PLL onto the entire substrate, though convenient, undermines the topographical cues of microridges, often leading to uncontrolled neurite outgrowth and branching [Figs. S3(b) and S3(c)]. Although PLL can be patterned onto the scaffold by a second photolithography process with removal of the photoresist afterward,^19,27^ this approach not only complicates the fabrication process but may also jeopardize the integrity of PLL and the stability of SU-8 features, as aggressive organic solvents are involved in the process. In contrast, our selective deposition method was simple and clean. By taking advantage of the liquid capillary forces, residual aqueous PLL solution aggregated at the corners of microridges after the polydimethylsiloxane (PDMS) slab was lifted off. Moreover, the hydrophobicity of the silicon nitride surface greatly facilitated the removal of the bulk PLL solution from the substrate. Considering the short interaction time, this process yielded minimum surface wetting and limited deposition of PLL onto unwanted background areas, while effectively coating PLL on the sidewall of SU-8 microridges [Figs. 1(c) and 1(d)].

**FIG. 2.**
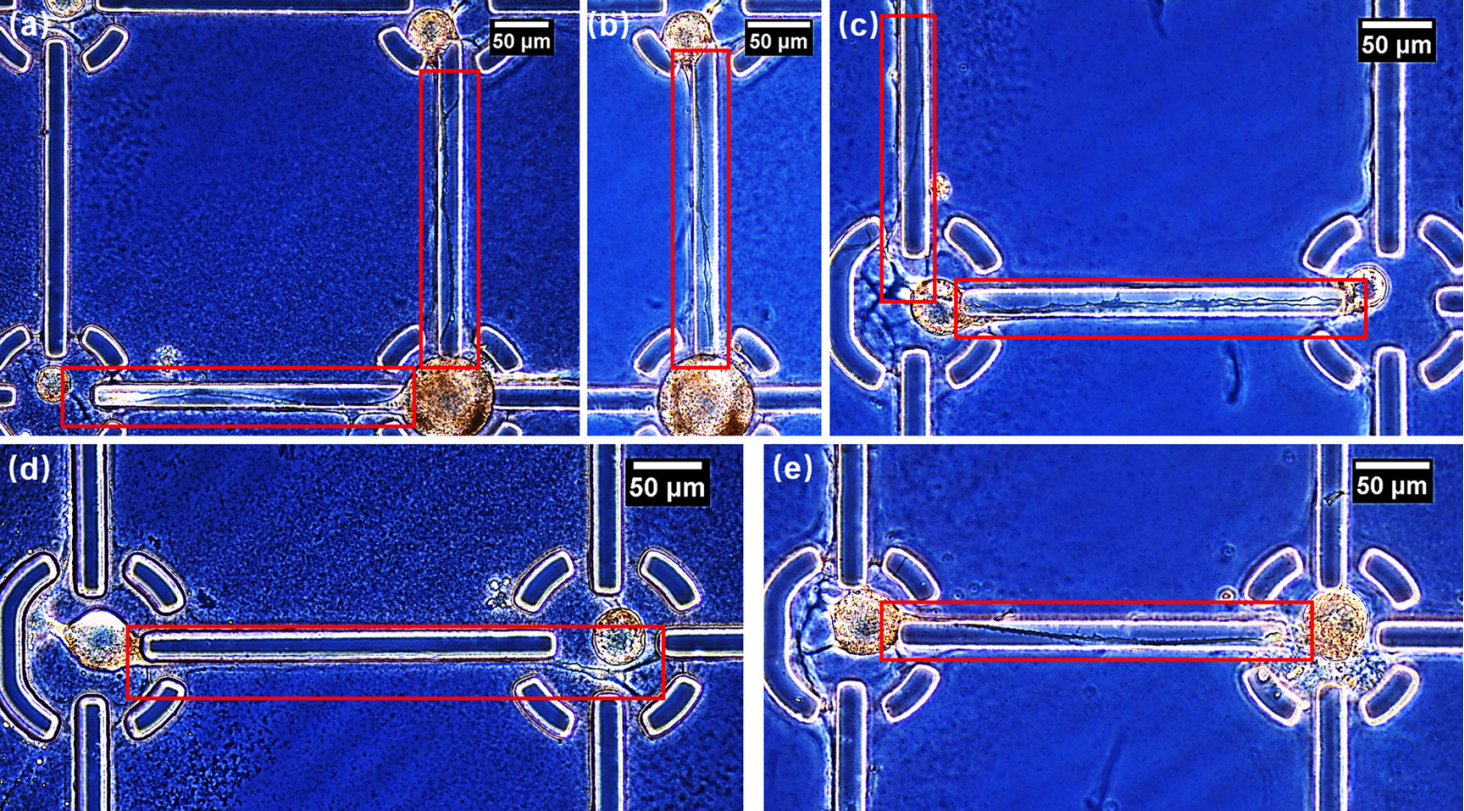
Phase-contrast microscopic images of guided *Aplysia californica* neurite outgrowth along microridges after 3 days of incubation. In (b), (c), and (e), regenerated neurites formed physical connections with neighboring neurons. In (d), the growth cone adhered to the SU-8 sidewall. In (a)–(c), and (e), the growth cone migrated from the sidewall at the axon stump to the topside.

The rationally designed microridge scaffold with selective PLL coating enabled *Aplysia californica* neurons to remain in their designated spots after cell seeding. Neurites were able to project along connecting lines toward adjacent cells (Fig. 2). These neurites adhered to the fiber-mimetic ridges for up to 600 *μ*m without substantial branching and deviation (Fig. S4). Although minor branching into undesired areas was occasionally observed, these deviated short neurites would likely not influence the circuit's function and might withdraw and degenerate if no connection was made.

**FIG. 3.**
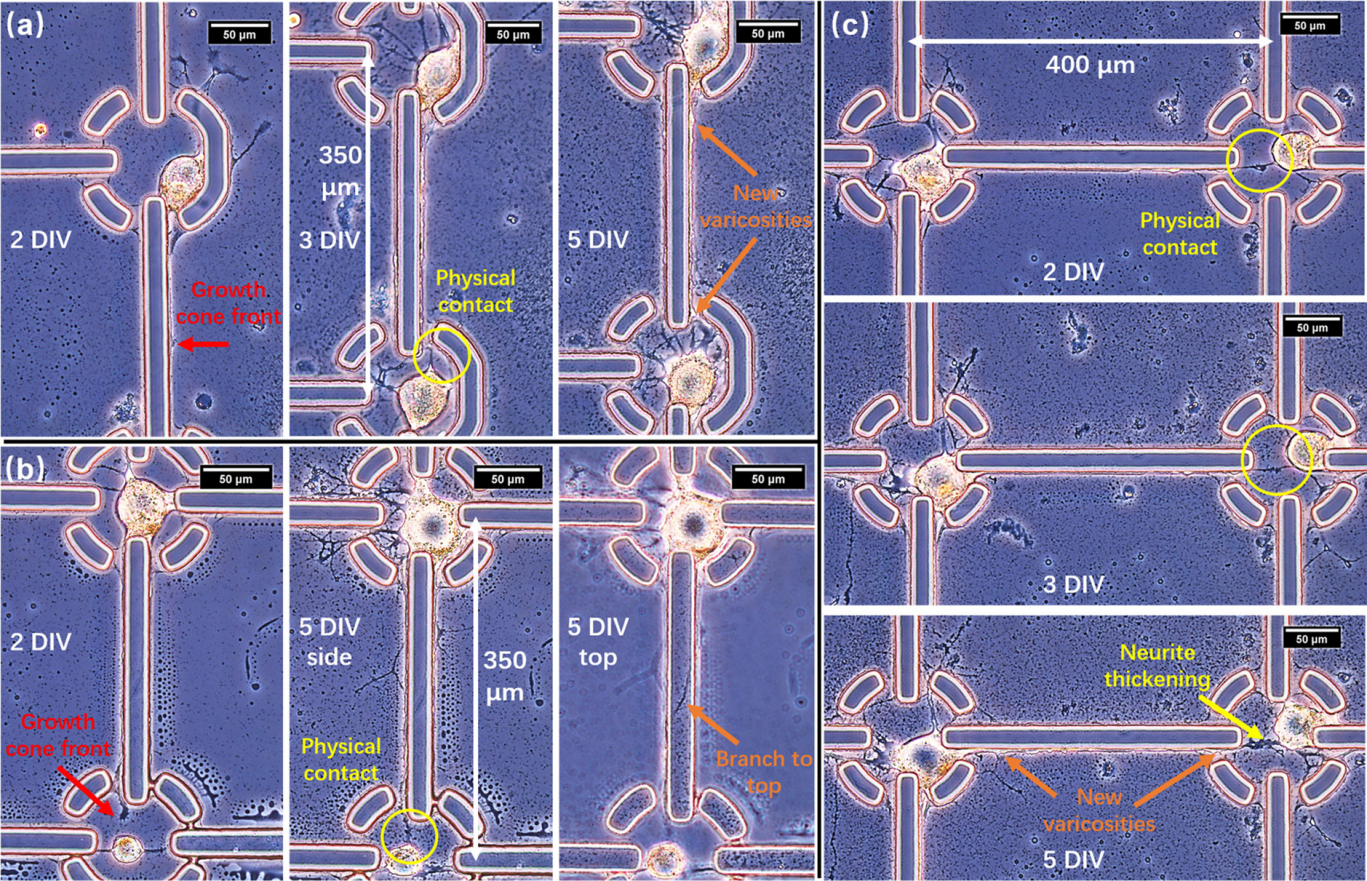
Process of neurite growth along microridges. The edges of microridges originally appeared smooth and relatively dark. Once neurites adhered to them, the edges became rougher (varicosities) and bright white-yellowish. (a) 350-*μ*m intercellular distance. Neurite from the upper cell progressed along the sidewall and formed physical contact with the lower cell on 3 DIV. The neurite became thicker with new varicosities on 5 DIV. (b) 350-*μ*m intercellular distance. Growth cone front from the upper cell reached the proximity of the lower cell on 2 DIV and formed physical contact on 3 DIV. We also observed that the neurite migrated from the sidewall to the topside of the microridge. (c) 400-*μ*m intercellular distance. Neurite from the left cell progressed along the sidewall and formed physical contact with the right cell on 2 DIV. The neurite became thicker with new varicosities on 5 DIV.

Nevertheless, when cells were separated with a 600-*μ*m distance, we observed that most growth cones stopped growing forward before reaching adjacent neurons. Therefore, we intuitively switched to shorter distances of 400 *μ*m and 350 *μ*m. As a result, more neurites were able to overcome the distance and reach adjacent cells. Although the mechanism is still unclear, in our understanding, shorter distances might assist the diffusion and sensing of neurotrophic factors released by adjacent neurons. Additionally, the neuritic trajectory was not always on the SU-8 sidewalls as expected but rather often migrated from side to top, or vice versa, exhibiting a semiwrapping-around behavior that might be unique to Aplysia neurons [Figs. 2(a)–2(c), 2(e), 3(b), and S5]. Considering the hydrophobicity of SU-8 and the resulted poor PLL coating on its top surface, this suggests that the microridge structure alone played an important role in maintaining the neurite's orientation in the absence of chemical cues. PLL might help provide an adhesive spot for filopodia at the early stage to initiate neurite outgrowth from the axon stump toward the terminal protrusions.

Morphological changes of neurite regrowth are shown in Fig. 3. Neuronal projections tipped with the growth cone, once adhered to the connecting line, steadily progressed forward during pathfinding [Figs. 3(a) and 3(b)]. After physical contact, neurites had shown increases in varicosities (visible bumps on neurites; see Fig. S6) and thickening [Figs. 3(a) and 3(c)]. To our understanding, this might be a suggestion of the formation of active synapses and change in synaptic efficacy that led to enhanced protein synthesis and transport along connected neurites.^48–50^ Note that “physical connections/contacts” here refers that the growth cone morphologically reached another neuron's soma or neurites, which is the prerequisite of formation of synapses but not a proof of either silent or active synapses. Such established connections could be self-maintained without deviation for at least 8 days (Fig. S5).

### Functional interneuronal communications

**FIG. 4.**
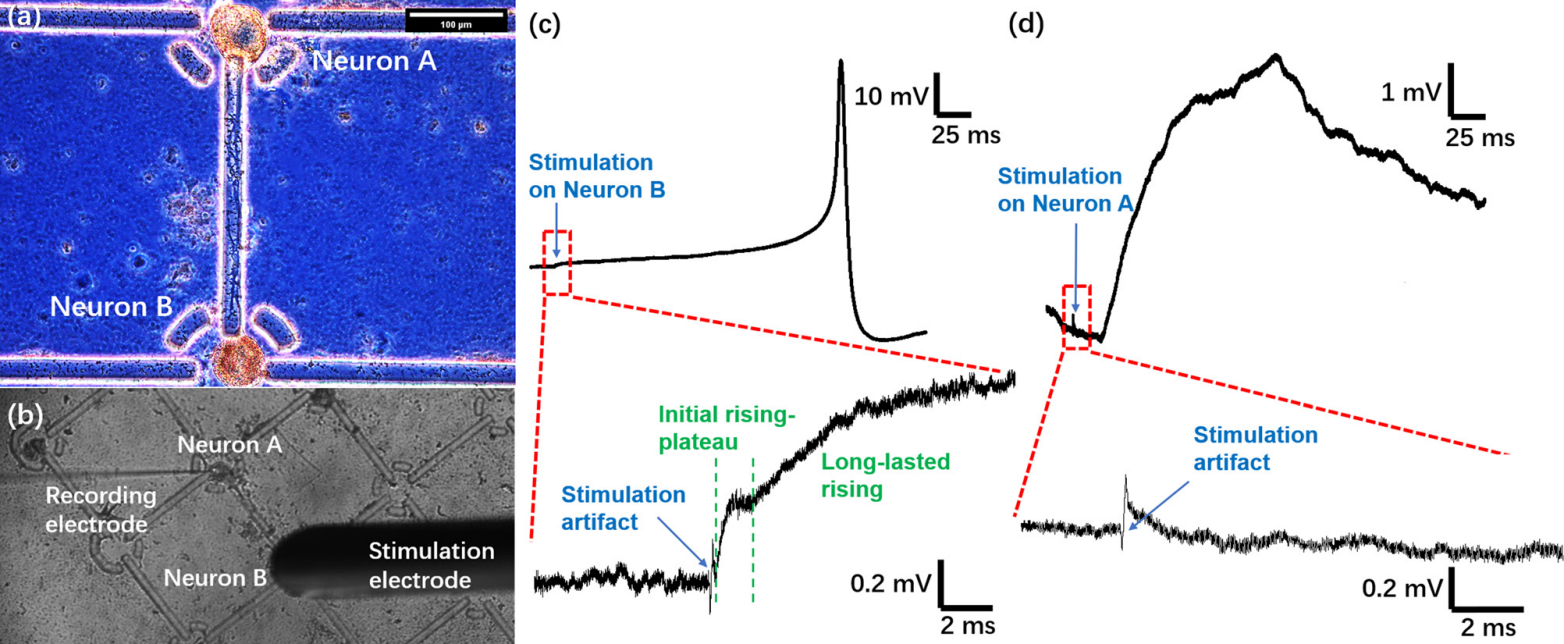
Electrophysiological test of functional wiring. (a) Morphologically wired Neurons A and B with 400-*μ*m distance. Neurites attached onto the topside of the connecting line. Scale bar: 100 *μ*m. (b) Photo showing the setup for the intracellular recording and extracellular stimulation. The recording electrode impaled Neuron A, and the stimulation electrode was placed right above Neuron B. (c) Membrane potential of Neuron A. Upper: an action potential was elicited 200 ms after a stimulation pulse on Neuron B. Lower: enlarged image of the red rectangle showing the stimulation artifact and the gradual depolarization afterward. (d) Membrane potential of Neuron B. Upper: an EPSP was elicited after a stimulation pulse on Neuron A with an about 20-ms delay. Lower: enlarged image of the red rectangle showing the stimulation artifact.

Besides accurate cell placement and morphological wiring, it is critical for neurobiological circuits to form functional synapses that enable intercellular signal transmissions. Therefore, we investigated the neuronal connections electrophysiologically to test if this platform was able to lead to formations of chemical synapses. For a pair of morphologically connected neurons [Fig. 4(a)], one cell was recorded by a penetrating intracellular electrode, while the other was stimulated extracellularly by a localized electrical pulse to elicit its action potentials [Fig. 4(b)].^46,47^ We did not use intracellular recording/stimulation on both cells because the 25-*μ*m fences often imposed a barrier difficult for both electrodes to properly penetrate the cells or caused bending and stress on the electrode tip that damaged the cell membrane. Since the concentric bipolar microelectrode had its own conductive shield, the stimulation pulse was only limited to the nearby cell without causing a large disturbance to other cells in the bath.

As shown in Fig. 4(c), after a stimulation pulse was delivered to Neuron B, the membrane of Neuron A was gradually depolarized over a 200-ms period, and eventually, an action potential was induced. This postsynaptic excitation was not a direct influence from the extracellular stimulus because both the amplitude (about 0.3 mV) and duration (about 0.1 ms) of the stimulation artifact were too small to impose enough membrane depolarization [Fig. 4(c), lower]. Moreover, we consistently observed a rising-plateau-rising profile after the stimulus [Figs. 4(c), lower, and S7], where the first rising-plateau phase (2 ms) could come from the extracellular shock and end with a plateau. The delayed and long-lasting depolarization afterward, however, was a typical characteristic of synaptic current injection. Additionally, during the 20 repeats of stimulation, an action potential was not invoked for every stimulus but rather with an interval of 1–3 repeats (Fig. S8), which suggested that the presynaptic terminals did not release enough neurotransmitters after a single stimulus, and accumulation of neurotransmitters over multiple stimuli triggered the firing of an action potential.

After confirming the synaptic connection from Neuron B to A, we switched the order and tested if there was reciprocal synaptic connection from Neuron A to B since *Aplysia californica* neurons, such as the L29 interneuron and LE (Left ganglion, cluster E) sensory neurons, can often form two-way connections.^51,52^ We recorded the intracellular membrane potential of Neuron B, while applying an extracellular stimulus to Neuron A. As a result, an EPSP was recorded in Neuron B with a synaptic latency of 20 ms from the stimulation artifact Fig. 4(d), which suggested that there was a reciprocal synaptic connection from Neuron A to B.

Note that since we did not perform intracellular recordings from presynaptic cells (for a dual patch clamp setup, microfences always blocked one of the sharp electrode's insertion path), the reason for large differences between Neuron A (action potential) and Neuron B (only EPSP) is not clear. We speculate that (1) Neuron B, under stimulation, fired a train of action potentials, which triggers Neuron A's action potential, whereas Neuron A fired only a single action potential that is enough for only EPSP in Neuron B; and/or (2) the synaptic strength of B (presynaptic) → A (postsynaptic) is significantly stronger than A → B; and/or (3) B → A has more excitatory synapses than A → B.

Moreover, due to the unavailability of synaptic staining antibodies (synaptophysin-eGFP and synapto-PHluorin,^53^ to efficiently tell whether synapses are structurally formed, before intracellular electrophysiology), we did not blindly repeat intracellular recordings on more neuronal pairs. This lack of comprehensive screening method also made it unclear how many pairs were synaptically connected in a 6 × 6 array. On the other hand, however, this might provide more flexibility to artificially choose which pairs to connect/disconnect, as electrical stimulation by MEA might significantly affect connectivity.

### Neuronal recording and stimulation with MEAs

Extracellular electrophysiology with MEAs is a useful tool for neuronal network analysis thanks to its parallel and noninvasive nature. The wiring microridges in this work can be readily fabricated on MEAs (Fig. S9), followed by the same capillary-coating approach. Soma of each single neuron was restricted by microfences to sit on top of each electrode site, maintaining stable cell-to-electrode registration.

Recording of spontaneous spikes was first carried out to test if such an integration could function properly. After 48 h culture at room temperature, neuronal spikes were detected (Fig. S10). The spikes had a bipolar shape with >100 *μ*V in amplitude and 1-ms spike duration (subfigure in Fig. S10), consistent with the typical extracellular spike waveforms. Although *Aplysia californica* culture showed a higher noise level than mammalian neurons, spikes could still be clearly identified by setting an appropriate detection threshold on the amplitude.

**FIG. 5.**
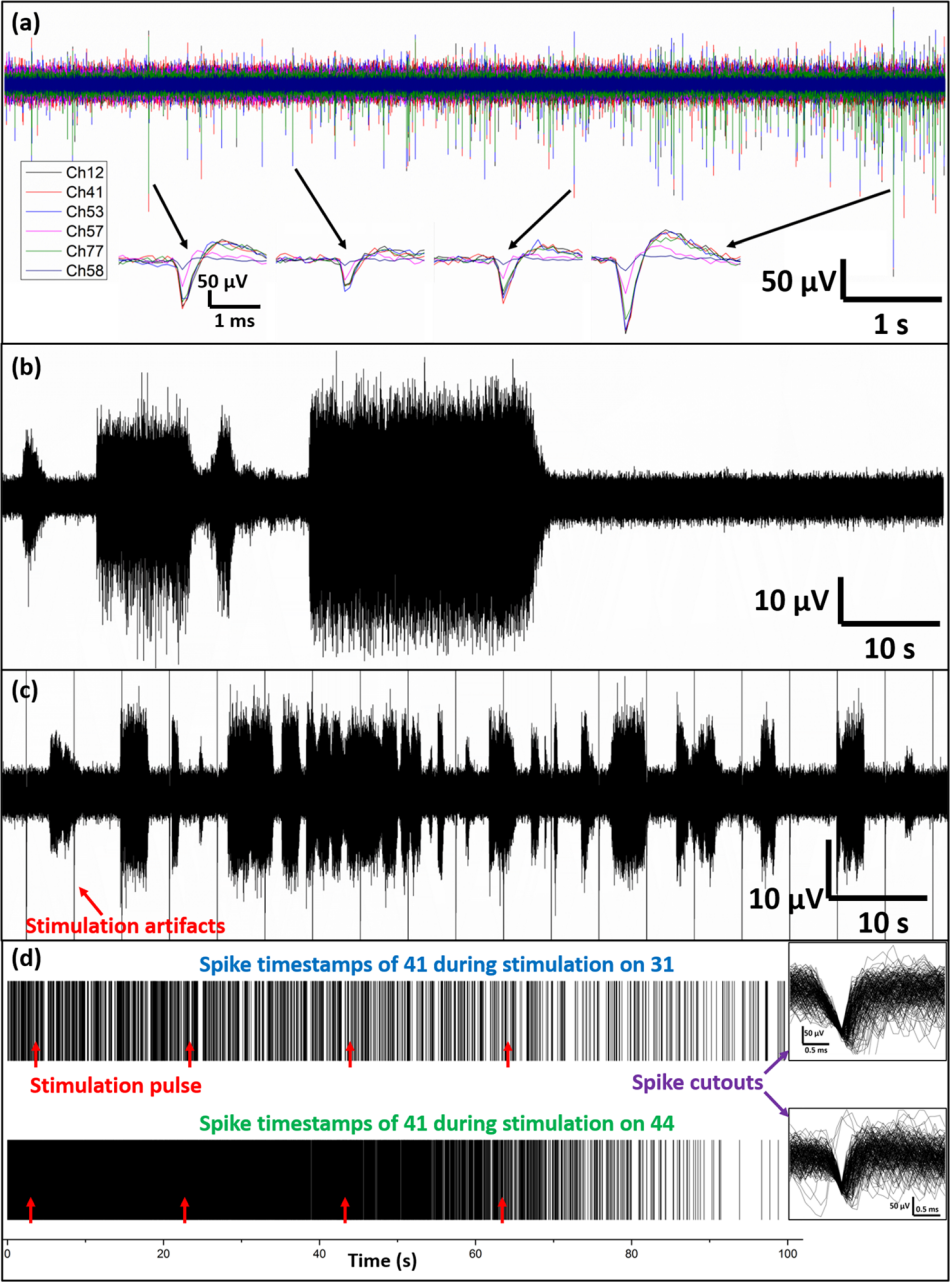
Spike recording and stimulation using microridge-MEA. (a) Synchronization of spike trains of neurons 12, 41, 53, 57, 77, and 58. The insets show that the peak and overshoot of each spike have a very short latency rather than being perfectly aligned. (b) Spontaneous bursting of neuron 42. (c) Stimulation-induced bursting of neuron 42 after 3 days from (b). (d) Spike timestamps of neuron 41, during stimulation on neurons 31 (upper) and 44 (lower), respectively. The red arrows denote the times of stimulation pulses. Insets on the right show the first 200 spike cutouts.

During spontaneous recording, we also observed spike train synchronization. As shown in Fig. 5(a), six neurons showed synchronized spike trains. The synchronized spikes were unlikely caused by passive transmission from one current source to others because (1) most of the electrodes did not record such synchronization (even for those closest to the synchronized neurons; see Fig. S11), (2) there was a latency between the peaks of spikes of different neurons [subfigure in Fig. 5(a)], and (3) neurons 57, 77, and 58 were no longer in synchronization with the others after 10 min (see Fig. S12; it indicated that the electrodes were not faulty and the changes came from neuronal interconnections). Due to the short interspike latency, it was likely that neurons formed direct electrical synapses through gap junctions or membrane fusion rather than chemical synapses (20-ms latency as shown in Fig. 4). Furthermore, the well-isolated spikes (not spreading to other electrodes) suggest that microfences, by providing lateral adhesion spots for the somata membrane (Fig. 3), might impose certain restriction on the leakage path of membrane currents. Such better sealed space between the cell membrane and electrodes increases the seal resistance.

Additionally, unique firing patterns may help us to interpret the identity of certain neurons. For example, we observed spontaneous bursting on neuron 42 and further recovered its bursting by electrical stimulation after 3 days [Figs. 5(b) and 5(c)]. Since only a limited number of cell types had been reported with bursting behavior (L10, R15, LUQ, and R25/L25),^54,55^ and considering its soma size smaller than the microfences, it was likely to be a neuron in the R25/L25 population that regulates the respiratory pumping in intact animals.

Finally, since each neuron was spatially registered to only one microelectrode, this platform might enable us to precisely induce activity-dependent adaption through electrical stimulation. Specifically, we first examined the neuronal network under a microscope and selectively labeled the subgroups in which neurons were interconnected morphologically by regenerated neurites. Then, electrical stimulation was applied to one cell, while other cells' responses were recorded. In the three-cell group of 31–41-44 (indicating the electrode number; see Fig. S9), 31–41 and 44–41 were visually interconnected neuronal pairs. As shown in the spike timestamp in Fig. 5(d), upon stimulation on cell 31, cell 41 showed a firing pattern relatively uniform throughout the recording period. In contrast, 41's firing activity gradually decreased when the stimuli were applied to cell 44. On the one hand, this might indicate that the 31-to-41 synapse was weak or not functional, while that of 44-to-41 was functionally interconnected. On the other hand, since pulses applied to neuron 31 did not alter neuron 41's firing pattern substantially, the contribution of stimulation artifacts could, therefore, be excluded from neuron 41's decreased firing rate (electrodes 44 and 31 were approximately equally distant to 41). Regarding neuron 41's decreased spiking activity, we speculate that under low-frequency stimuli, neural adaption was induced at the 44–41 synapse due to neurotransmitter depletion, which led to the adaptive responses in neuron 41 to repetitive stimulation.

Our microridge guiding platform can be well integrated with MEAs for neural recording, stimulation, and modulation in a precise manner. Although we showed a few examples to demonstrate its potential application in functional neural circuits, it should be noted that (1) this current work provided a convenient platform for fabricating accurate neuronal circuits rather than actually built a fully functional multicellular circuit; and (2) characterization on the circuit variation (same type of cells in different samples) and time variation (same sample on different time points) is critical to understanding neuronal behaviors in artificially organized constructs, yet it needs to be carried out in the future; and (3) understanding the exact mechanisms of neuronal connections and constructing rationally designed neuronal circuits certainly require much more comprehensive efforts, such as robust surgical harvest of individual cells, live-cell Ca^2+^ imaging, and large-scale spike data processing, which are far beyond the scope of this current work.

## DISCUSSION

A neuron, as the fundamental computational unit of the nervous system, is precisely connected to its targets *in vivo* and serves a unique function during information processing. Networks with hundreds to billions of these wired neurons function as neurobiological circuits to control animals' responses to various environmental stimuli. However, the lack of neural heterogeneity and fabrication approaches are two major obstacles for reconstructing functional neurobiological circuits *in vitro*, as compared to electronic circuits. Despite extensive investigations on the structure and function of *in situ* neural circuits, massive cell harvesting and plating often result in random neuronal networks with an unspecified structure and function. Because of this technical gap, it is difficult to precisely reconstruct a known neurobiological circuit *in vitro*. This current effort aimed to address these issues by integrating precise cell positioning and microfiber-mimetic guidance into neurobiological circuit fabrication.

Our integrative approach of micropatterned ridges and cell adhesion molecules offered precise 2D patterning of neuronal networks. Inspired by neurons' natural affinities to microfiber structures, this approach anchored cells one-by-one in designated positions, while enabling their neurites to grow toward target cells through guided pathfinding. Although nondirected neurite outgrowths were occasionally observed, with appropriate intercellular distances, the slowly growing, deviated neurites did not interfere with our rationally designed circuits. From our cell matrix test with 400-*μ*m intercellular distance, among the 13 neurons with outgrowth, 10 of them had growth cones well guided by SU-8 lines (no deviations or very limited deviations <50 *μ*m), two of them were observed with guided growth cones but had noticeable branches (50 *μ*m < deviation < 200 *μ*m), and one of them had no neurite guided (Fig. S13; here, we solely report our best result, which is not a comprehensive evaluation). Neuritic physical contacts only occurred between guided neurites, and the deviated branches had made no physical contacts. However, since *Aplysia californica* neurons have strong tendency of neurite branching,^56^ an intercellular distance smaller than 300 *μ*m causes neurites to significantly deviate from the guiding microridges. We suspect that it was because neurotrophic factors released from adjacent cells imposed stronger guiding effects than the artificial cues from microridges and PLL. Overall, the intercellular distance is an important factor affecting the yield and deviation of neurite connections. However, in this current work, confidently quantifying the effect of the intercellular distance is still limited by (1) the low cell number in each batch (6 × 6) and (2) unpredictable cell death during surgical isolation, transfer, and plating.

Furthermore, since this scaffold can be easily integrated with MEAs, functional electrical stimulation might be used to (1) modulate the circuit wiring while cell-cell connections are formed and (2) program the circuit function after construction. Electrical recordings can be used to monitor the internal states of the circuit. For example, delivering paired electrical stimuli may facilitate the formation of synapses between aligned neurites, and employing activity-dependent plasticity^57,58^ could induce neurite retraction and removal of unwanted connections. However, although we integrated microridges with MEAs for simple recordings and stimulations, more thorough work is still needed to fully investigate what stimulation protocols could induce short- and long-term synaptic plasticity between precisely wired neurons to realize circuit-like functions.

Conventionally, the SU-8 features in this work are not considered as typical fibers that are flexible and freestanding or being spun woven. However, this design was motivated by the neurons' natural tendency to migrate along fiberous structures like radial glia fibers. On a 2D plane, the SU-8 features do not have much topographical difference than a lie-down nylon fiber (Fig. S1). Moreover, regenerated neurites could often migrate from the sidewall to the top, and vice versa, exhibiting a wrapping-around behavior rather than adhering to the sidewalls all the time or crossing over the ridge to the unwanted silicon nitride area. This further confirms that microridges have similar effects to lie-down fibers on neurite pathfinding.

It is also worth mentioning that *Aplysia californica* neurons were chosen here simply for a demonstration purpose because of the ease of cell manipulation. Mammalian neurons with smaller diameters (around 10 *μ*m) will be challenging to manually seed with glass micropipettes, though the patterns and dimensions of the scaffold can be easily scaled down to thickness less than 5 *μ*m. However, with the advances of single cell manipulation technologies (e.g., glass micropipette assisted using a miBot micromanipulator; Imina Technologies SA, Switzerland),^59^ this approach holds a great promise for precisely constructing neuronal circuits using mammalian neurons.

## CONCLUSIONS

We presented an effective approach for precise fabrication of rationally designed 2D neuronal circuits. The advantages of this method are as follows: (1) plating individual neurons with rationality and immobilizing somas at designated spots, (2) well-controlled growth cone by microfiber-like guidance utilizing neurons' natural affinities, and (3) single-layer lithography and capillary force coating being convenient for fast prototyping of neuronal circuit designs. Following the cues imposed by selectively PLL-coated SU-8 features, neurites could be guided to grow in designated directions for at least 600 *μ*m without substantial deviations. Reducing the intercellular distance to 400 *μ*m and 350 *μ*m facilitated neuronal connections both physically and functionally through chemical synapses, allowing the transmission of action potentials and subthreshold synaptic potentials. Together with integration compatibility with MEAs, this approach holds great promise for precisely constructing neuronal circuits that can be used in both fundamental research and clinical applications.

## METHODS

### Microridge platform fabrication

**FIG. 6.**
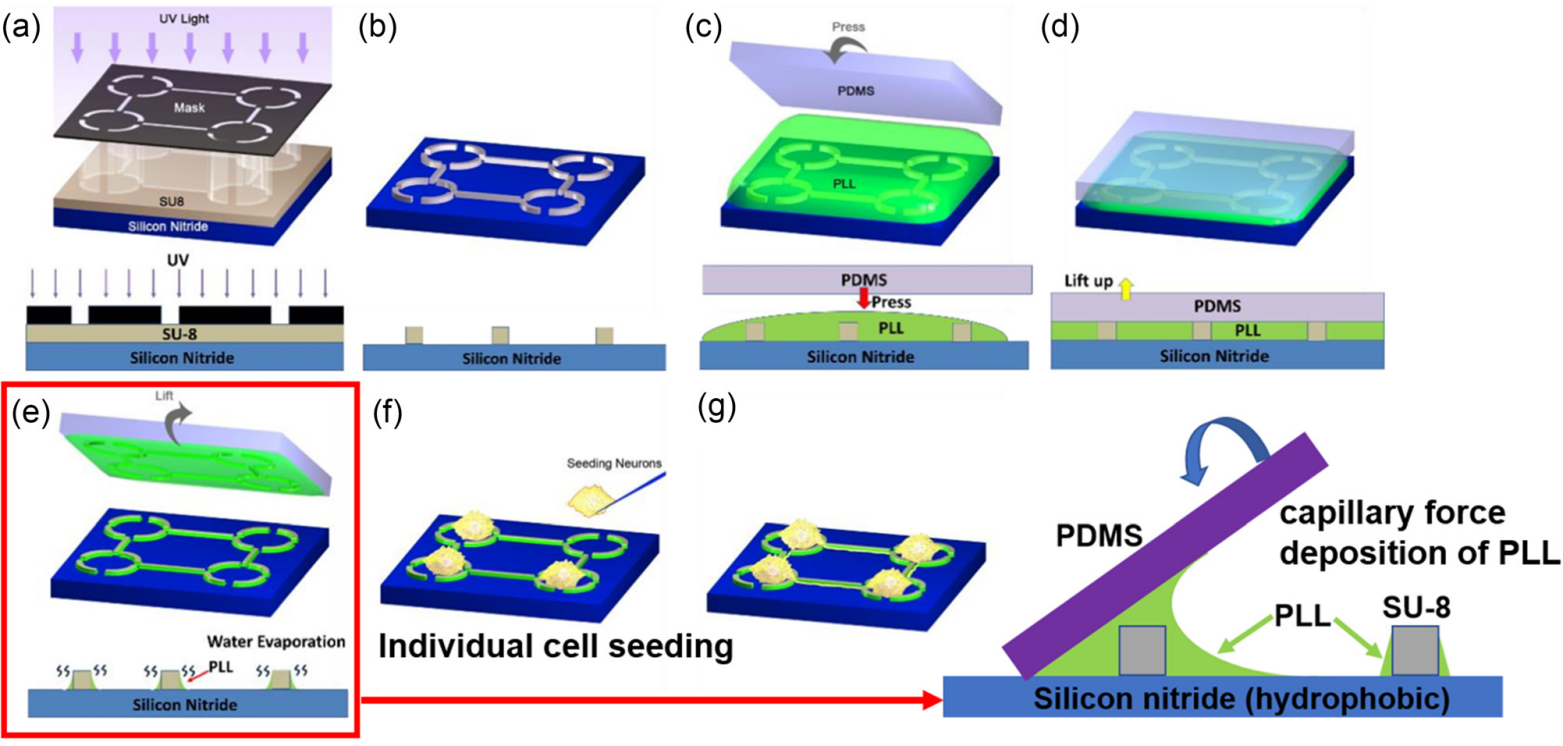
Illustration of the fabrication, coating, and cell seeding processes of an SU-8 microridge scaffold. (a) and (b) Fabrication of microfences and connecting lines through one-layer photolithography. (c) Microstructures were covered by an aqueous PLL solution, and a PDMS slab is, then, placed on top. (d) The PDMS slab was lifted off to remove the majority of PLL solution on the hydrophobic silicon nitride surface. (e) Capillary forces retained PLL solution residues in the corners of microridge sidewalls. PLL was selectively deposited onto the sidewalls after water was evaporated. (f) *Aplysia californica* neurons were manually seeded in the microfences one by one using prepulled glass micropipettes. (g) Guided neurites grown out toward neighboring neurons.

The microridge scaffold was fabricated on a silicon nitride surface through single-step photolithography [Figs. 6(a) and 6(b)]. Briefly, SU-8 2025 photoresist (MicroChem) was spin-coated at 3000 rpm onto a silicon nitride surface (PECVD deposited) on a 25 mm circular glass coverslip and soft baked at 65 °C and 95 °C sequentially, yielding a 25-*μ*m film. Photolithography was, then, carried out by exposing the SU-8 film with filtered i-line UV light through a soft photomask (CAD/Art Services). After post exposure baking at 65 °C and 95 °C, the sample was developed in the SU-8 developer for 150 s and rinsed with isopropanol, producing microridges of 20 *μ*m in width and 25 *μ*m in height. Reconstructed 3D topographical profiles were acquired using Zeta-20 optical profiler (ZETA Instruments).

The same photolithography process was also applied on a custom-made MEA, with an additional step of aligning the circular SU-8 fences with microelectrodes.

### Selective deposition of PLL with capillary force

We used a simple press-and-lift strategy to selectively coat PLL onto the sidewalls of microridges by exploiting the effect of surface tension [Figs. 6(c)–6(e)]. The substrate was first sterilized with 70% ethanol for 30 min and then rinsed with deionized (DI) water. After drying, a droplet of PLL solution (Sigma, 0.5 mg ml^−1^ in 0.1 M Na Borate buffer) was dripped onto the substrate, covering the patterned area. Then, a plain PDMS slab was pressed against the patterned area to squeeze out extra solution. Immediately, the PDMS slab was carefully lifted off. As a result, the majority of the PLL solution on the silicon nitride surface was taken away by the PDMS slab, with the residue only left in the corners of microfeature sidewalls due to capillary forces. The device was, then, left in a biosafety cabinet for 30 min to evaporate water from the residual solution, leaving PLL molecules deposited onto the sidewalls. To examine the selective deposition of PLL, the same procedures were conducted with fluorescein isothiocyanate (FITC)-labeled PLL (Sigma, 0.2 mg ml^−1^), and the samples were checked under a fluorescent microscope (EVOS, Life Technologies).

### Culture of *Aplysia californica* neurons

Ethical approval is not required for *Aplysia californica* cell harvest in this study. Isolation and maintenance of *Aplysia californica* neurons had been described previously.^44^ Briefly, abdominal ganglia from 5 to 20 g animals were incubated in a 10 units ml^−1^ protease solution (Sigma) for 150 min. Meanwhile, the microridge scaffold device was immersed in the culture medium (50% *Aplysia californica* hemolymph and 50% Leibovitz's L15 medium (Gibco), with salt concentrations of 400 mM NaCl, 11 mM CaCl_2_, 10 mM KCl, 27 mM MgSO_4_, 27 mM MgCl_2_, 2 mM NaHCO_3_, 6 mg ml^−1^ dextrose, and 0.1 mg ml^−1^ L-glutamine^45^). After incubation, the digested ganglia were transferred into a Petri dish, where cells were dissected and transferred onto the microridge scaffold by hemolymph coated micropipette tips. Using prepulled glass micropipettes (Sutter Instruments), individual cells were placed one-by-one onto designated spots surrounded by the microfences [Figs. 6(f) and 6(g)]. A 6 × 6 cellular matrix was used for cultures without MEA, and the cultures with MEA have 59 cells. The cell culture was ready for observation after 24 h incubation at 18 °C.

### Electrophysiology

Electrophysiology tests were conducted according to previously reported procedures.^46,47^ After 5 days in culture, synaptic connections of paired neurons were tested. One cell was penetrated by a sharp glass microelectrode (26 MΩ, filled with 2.5 M KCl) for intracellular recording, while the other was extracellularly stimulated by a 75-*μ*m diameter concentric bipolar electrode (Frederick Haer Company, Bowdoinham, ME, USA). The far-away bath was grounded by a large Ag/AgCl electrode. Stimulation pulses (0.1 ms, 8 V, 500-ms interval, 20 repeats) were applied between the electrode's inner pole and outer stainless steel shield. At 307 ms of each repeat period, a hyperpolarizing current (100 ms, −100 pA) was injected into the cell under recording to recover its resting potential. Then, the recording and stimulation were reversed on the two neurons to test if there were reciprocal synaptic connections. EPSPs were recorded under current-clamp mode, and the electrode was held at −60 mV during the impalement of the neuron. The tests were performed with pClamp10 software, the Digidata 1550 acquisition system, and a Multiclamp 700B amplifier (Axon Instruments, Union City, CA, USA).

Extracellular electrophysiology was conducted on a custom-made MEA whose electrodes (30 *μ*m in diameter) were electrodeposited with Pt black. The MEA was designed to be compatible with the MEA2100 acquisition system (Multichannel Systems). A Ag/AgCl wire was immersed in the bath as ground. Neurons were plated one by one at each electrode, with a total of 59 cells (60 channels—1 reference channel). For spike recording, signals were sampled at 10 kHz and high-pass filtered at 200 Hz to remove the local field potentials. The acquired data were further processed using the MultiChannel Analyzer software for spike detection. The threshold was manually set to −100 *μ*V, and the time window for spike cutouts was set to 3 ms. For electrical stimulations, single bipolar pulses (±50 mV, 20 ms) with an interpulse interval of 20 s were applied on selected microelectrodes. Circuit blanking was applied to avoid stimulation artifacts on nearby electrodes.

## SUPPLEMENTARY MATERIAL

See the supplementary material for the neurite deviations, varicosities, physical contacts, additional electrophysiology information, and cell matrix results.

## AUTHORS' CONTRIBUTIONS

Y.W. and M.W. contributed equally to this work.

## Data Availability

The data that support the findings of this study are available within the article and its supplementary material.
